# Editorial: Reviews in elite sports and performance enhancement

**DOI:** 10.3389/fspor.2023.1353575

**Published:** 2024-01-05

**Authors:** Olivier Girard, Chris Bishop

**Affiliations:** ^1^ School of Human Sciences (Exercise and Sport Science), The University of Western Australia, Perth, WA, Australia; ^2^ London Sport Institute, Middlesex University, StoneX Stadium, London, United Kingdom

**Keywords:** global publishing trends, reviews articles, evidence-based practice, research impact, elite sport

**Editorial on the Research Topic** Reviews in elite sports and performance enhancement

## Introduction

The current global publishing trend has witnessed a simultaneous rise in both original research papers and literature reviews. The expansion of evidence-based practice has notably resulted in a growing variety of review types in recent years. It is indisputable that the COVID-19 pandemic has significantly impacted various aspects of life, including the publishing landscape. In sports science, its effects on the volume of scholarly publications, particularly the number of review papers, have been substantial, notably between 2020 and 2022. This can be attributed in part to disruptions in access to laboratories, gyms, and pitches, allowing practitioners more time to undertake work-related activities that were often not prioritised. This Research Topic aims to highlight recently published “Reviews in Elite Sports and Performance Enhancement”.

## This research topic

An umbrella review consolidates evidence from multiple reviews into a single document, addressing a broad condition or problem with competing interventions. Based on the 14 meta-analyses and the 178 primary studies in their umbrella review, Bernárdez-Vázquez et al. provide valuable information for the design and configuration of resistance training programs aiming to optimize muscle hypertrophy. Key findings include optimal effectiveness with a minimum of 10 sets per week per muscle group, the importance of eccentric contractions, avoidance of very slow repetitions (≥ 10 s), and potential benefits of blood flow restriction training for some. Conversely, variables such as exercise order, time of day, and periodization type were not found to directly impact muscle mass gains. Consequently, these insights are valuable for optimizing resistance training programs.

The second paper in this collection continues on the topic of strength and conditioning. In their narrative review, Warneke et al. address the common use of isometric testing as an alternative to dynamic strength assessment. The review emphasizes that high correlation coefficients (*r* ≥ 0.7) between the two tests do not guarantee their interchangeability. Instead, it recommends using the concordance correlation coefficient and Bland-Altman analysis, including mean absolute error and the mean absolute percentage error, to assess replaceability. Overall, this narrative review underscores the limited validity of correlation coefficients and suggests that a mean absolute percentage error of around 17% as intolerably high for comparing testing procedures.

Unlike narrative reviews, a systematic review seeks to systematically search for, appraise, and synthesize research evidence, often adhering to guidelines on the conduct of a review (1). Using this methodology, Poteko et al. explored the impact of the social disruption caused by the COVID-19 pandemic on various aspects of elite athletes’ lives from: micro- (individual), meso- (organizational), and macro-social (national and international) perspectives. Through an inductive coding process, the review's key findings cluster around three themes: (a) Inequalities—highlighting disparities exacerbated by the pandemic; (b) Crisis management—identifying key factors for consideration during social disruptions; and (c) Opportunities—viewing the pandemic as a chance for transformative changes in sport. Overall, it highlights the disruption of established routines and the exacerbation of pre-existing inequalities by the pandemic.

Another systematic review examined coping strategies used by elite athletes to manage stress and their impact on mental health. The study by Nuetzel, the first of its kind, identifies a diverse range of coping strategies reflecting athletes’ coping styles. The application of coping strategies appears linked to the type and intensity of stressors and the athlete's cognitive appraisal. Results emphasize the crucial role of coping strategies in handling both sport-specific and non-sport-specific stressors in an athlete's career. While evidence supports the effectiveness of coping in mitigating stress, the review notes a lack of intervention-based study designs, making the interrelationships between stressors, coping strategies, and mental health outcomes still unclear.

Systematic reviews incorporating a meta-analysis component employ statistical techniques to synthesize data from multiple studies into a single quantitative estimate or summary effect size. Using this approach, Han et al. identified significant differences in height, body mass, agility, speed, endurance capacity, and lower body power between elite and non-elite basketball players. The results of this meta-analysis highlight the intricate relationship among anthropometric, physiological, and physical performance factors. Notably, these factors may influence early basketball development, providing advantages in court coverage and reaching the basket. Additionally, early exposure to activities building endurance and lower body strength appears to be crucial. The conclusion emphasizes the interplay of talent, physical attributes, and skill acquisition in maximizing the potential for young basketball players.

Scoping reviews identify and examine characteristics or factors related to a particular concept. The last paper in this collection questions the place that elite sport holds for women. In their two-part study involving a sociohistorical analysis and a scoping review of existing sport science literature using Newell's constraints-led approach, Fraser and Kochanek emphasize the persistent reliance on male-centric evidence to date, despite increased attention and investment in female sports. The review reveals a gap, as none of the ten identified studies focus on female athletes or sociocultural constraints. The discussion advocates for an integrative, interdisciplinary approach and a departure from implementing male-centric evidence in female athletes. Notably, it urges stakeholders to recognize potential differences in female athletes, promoting gender equity in sports.

## Moving forward

**Figure 1:**
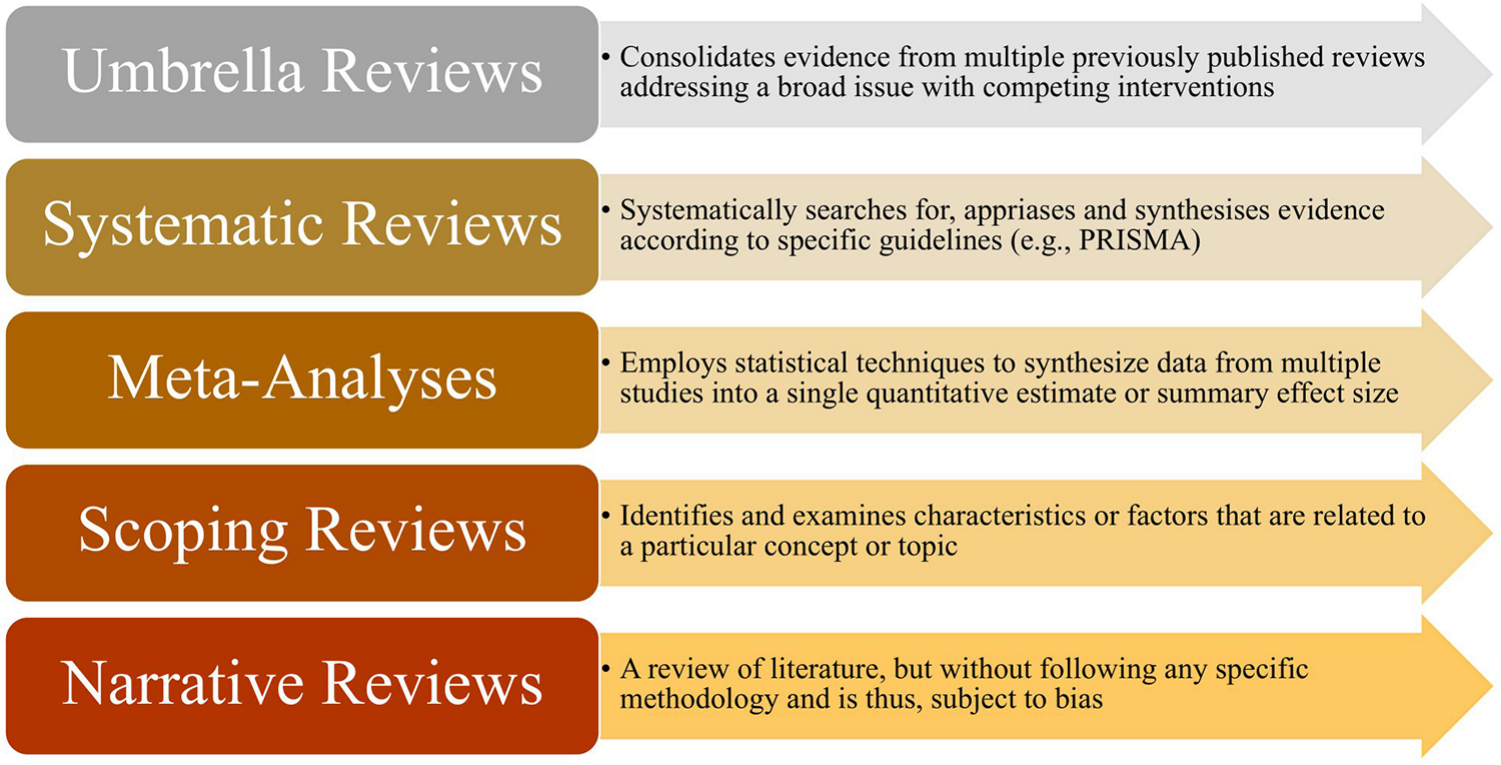
Schematic overview of the different kinds of review articles available in this research topic.

The academic publishing landscape is undergoing significant changes, marked by a steady rise of annual publications, including a growing number of review papers. The diverse array of review papers, in terms of scope and methodology, within this Research Topic underscores the valuable contribution of the Elite Sports and Performance Enhancement section of *Frontiers in Sports and Active Living* (Figure 1). With its 16 distinct sections, this journal stands out for its unique promotion of multidisciplinary research in sport science, exercise, and health. This platform serves as a means to divert from siloed thinking, aiming to strengthen the overall capacity of the sport science community. By embracing this approach, we expect that innovative thinking will drive advancements in concepts, best practices, and technologies within the realm of rehabilitation, prevention, and athletic training. This may notably facilitate a deeper understanding of how factors such as climate change, modern hectic lifestyles, and inequality impact athletes and active populations.
